# Retrospektiver Vergleich der roboterassistierten und der laparoskopischen Pyeloplastik an zwei Zentren

**DOI:** 10.1007/s00120-020-01414-3

**Published:** 2020-12-08

**Authors:** H. Griessner, L. Oberhammer, M. Pallauf, D. Oswald, T. Kunit, D. Colleselli, A. Merseburger, M. Kramer, L. Lusuardi, M. Mitterberger

**Affiliations:** 1 Universitätsklinikum der Paracelsus Medizinischen Privatuniversität, Universitätsklinik für Urologie und Andrologie, Landeskrankenhaus Salzburg, Müllner Hauptstraße 48, 5020 Salzburg, Österreich; 2 Campus Lübeck, Klinik für Urologie, Universitätsklinikum Schleswig-Holstein, Lübeck, Deutschland

**Keywords:** Nierenbeckenplastik, Roboterassistiert, Laparoskopisch, Anderson-Hynes, Nierenbeckenabgangsstenose, Pyeloplasty, Robotic assisted, Laparoscopic, Anderson-Hynes, Renal pelvic outlet stenosis

## Abstract

**Ziel:**

Wir verglichen in unserer retrospektiven Multicenterstudie die Ergebnisse der konventionell laparoskopischen Nierenbeckenplastik (L-NBP) mit denen der roboterassistierten Nierenbeckenplastik (R-NBP) nach Einführung des da Vinci X-Systems.

**Methoden:**

Insgesamt wurden im definierten Zeitraum von Mai 2015 bis September 2019 76 Nierenbeckenplastiken an zwei unterschiedlichen Universitätskliniken durchgeführt. Für die Datenanalyse wurden 63 Patienten berücksichtigt, welche entweder eine L‑NBP (*n* = 27) oder eine R‑NBP (*n* = 36) nach Anderson und Hynse erhielten.

**Ergebnisse:**

Das mediane Follow-up lag bei 22,5 (L-NBP) bzw. 12,7 (R-NBP) Monaten. Die statistische Analyse der Patientengruppen ergab bzgl. Alter, BMI, Geschlecht und betroffener Seite keinen statistischen Unterschied. Die Operationszeit war in der Gruppe der R‑NBP nicht statistisch signifikant kürzer (180 ± 72 vs. 159 ± 54 min, *p* = 0,194). Bezüglich postoperativer Major- bzw. Minor-Komplikationen nach Clavien-Dindo, Krankenhausaufenthaltsdauern (7,48 ± 2,86 vs. 6,33 ± 2,04 Tage) und Erfolgsrate ergab sich ebenso kein statistisch signifikanter Unterschied.

**Schlussfolgerung:**

Unsere Daten zeigen keinen signifikanten Unterschied der beiden Gruppen bezogen auf die peri- und postoperativen Ergebnisse. Es konnte gezeigt werden, dass für den Patienten auch unmittelbar nach Implementierung eines robotischen Systems kein Nachteil entsteht.

## Abstract (en)

**Purpose:**

The aim of this retrospective study was to compare our initial experience with robotic assisted laparoscopic pyeloplasty (R-LPP) with the conventional laparoscopic method (C-LPP).

**Materials and methods:**

In the defined period from May 2015 to September 2019, a total of 76 renal pelvic surgeries were performed at two different university clinics. In all, 63 patients who received either L‑NBP (*n* = 27) or R‑NBP (*n* = 36) were considered for data analysis.

**Results:**

The median follow-up for C‑LPP and R‑LPP was 22.5 and 12.7 months, respectively. The statistical analysis of the two groups revealed no statistically significant difference regarding age, body mass index, gender or affected side. The operating time was nonsignificantly shorter in the R‑LPP group (180 ± 72 vs. 159 ± 54 min, *p* = 0.194). There were no statistically significant differences in postoperative pain, complications, average length of stay in hospital (7.48 ± 2.86 vs. 6.33 ± 2.04 days) or success rate.

**Conclusion:**

This study shows no significant reduction in operating time in the R‑LPP group with an equal rate of complications. It could be shown that there is no disadvantage for the patients undergoing R‑LPP directly after the implementation of a robotic system.

In den letzten 10 Jahren wurde die Nierenbeckenplastik zunehmend robotisch durchgeführt und löste die offene und die konventionell laparoskopische Technik weitgehend ab. Durch die Einführung der roboterassistierten Laparoskopie ergibt sich eine Vielzahl potenzieller Vorteile, wodurch die Lernkurve des Operateurs wesentlich verbessert werden kann [14, 17]. Im folgenden Beitrag wird ein Vergleich der konventionell laparoskopischen (L-NBP) mit der roboterassistierten Nierenbeckenplastik (R-NBP) unmittelbar nach Einführung des da Vinci X-Systems präsentiert.

Bei der Nierenbeckenabgangsenge besteht ein erhöhter Widerstand für den Urinfluss, wodurch es zu einer chronischen Drucksteigerung im Nierenbecken kommt. Intrinsische Obstruktionen, Harnleiter-Kinking, Strikturen oder extrinsische Kompressionen, z. B. durch ein kreuzendes Unterpolgefäß, stellen die häufigsten Ursachen für eine Nierenbeckenabgangsstenose dar [16].

Traditionellerweise war die offene Nierenbeckenplastik der Goldstandard bei der Behandlung der Nierenbeckenabgangsstenose. 1949 beschrieben Anderson und Hynes die offene Nierenbeckenplastik, welche heute noch die Grundlage der L‑NBP bzw. R‑NBP ist [1, 8]. Diese Methode bedingt die komplette Exzision der anatomischen Engstelle sowie die Entfernung des überschüssigen Nierenbeckens. Vor allem das laparoskopische Nähen der Anastomose benötigt viel Zeit und erfordert ein großes Maß an Erfahrung. Durch die Einführung der roboterassistierten Laparoskopie ergibt sich eine Vielzahl an potenzieller Vorteile wodurch komplexe Arbeitsschritte wesentlich schneller durchgeführt werden können [14, 17].

Wir verglichen in unserer retrospektiven Multicenterstudie die Ergebnisse der konventionellen L‑NBP mit denen der R‑NBP nach Einführung des da Vinci X-Systems. In den meisten Studien werden zwei etablierte Systeme miteinander verglichen. Die Frage, ob Patienten unmittelbar nach Umstellung eines etablierten Systems benachteiligt sind, wurde bisher kaum untersucht. Ziel dieser Studie ist zu zeigen, dass nach Einführung des da Vinci X-Systems bereits vergleichbare peri- und postoperative Ergebnisse erzielt werden und kein Nachteil für den Patienten entsteht.

## Material und Methoden

### Patienten

In dem definierten Zeitraum zwischen 2015 und 2019 wurden insgesamt 76 Patienten Nierenbeckenplastiken durchgeführt, welche 30 Monate vor bzw. 20 Monate nach Einführung des da Vinci-Systems an der urologischen Abteilung des Universitätsklinikums Salzburg bzw. an der urologischen Abteilung des Universitätsklinikums Schleswig-Holstein operiert wurden. In diesem Zeitraum wurden 6 offene bzw. 7 Fenger-Plastiken durchgeführt, für die weitere Analyse wurden jedoch nur R‑NBP bzw. L‑NBP nach Anderson und Hynes berücksichtigt.

In diesem Zeitraum wurden 27 dieser Patienten konventionell laparoskopisch operiert, 36 Patienten der Vergleichsgruppe wurden roboterassistiert operiert. Die erste R‑NBP erfolgte in Schleswig-Holstein im November 2017 und in Salzburg im Dezember 2017. Die Diagnose der Nierenbeckenabgangsstenose wurde mittels Nierenszintigraphie (dynamische Nierenfunktionsszintigraphie mit 133 MBq Technetium 99 MAG‑3 und Lasix-Belastung) bzw. bei symptomatischen Patienten mittels retrograder Pyelographie im Rahmen der Harnleiterschienenanlage gestellt.

Die Nachsorge der Patienten erfolgte je nach Patientenwunsch über die jeweiligen Krankenhäuser bzw. durch Urologen im niedergelassenen Bereich. Zur Beurteilung der Langzeitergebnisse wurden Daten aus dem jeweiligen Krankenhausinformationssystem bzw. der behandelnden Urologen berücksichtigt.

Das neuerliche Vorliegen einer symptomatischen Nierenbeckenabgangsenge bzw. der Nachweis einer relevanten Obstruktion in der Nierenszintigraphie wurden als Rezidiv definiert.

### Operationstechnik

Lediglich Patienten mit einer symptomatischen Nierenbeckenabgangsstenose (Schmerzen, steigende Nierenfunktionsparameter bzw. akute Nierenbeckenentzündung) erhielten präoperativ einen Harnleiterstent. Bei allen eingeschlossenen Patienten wurde eine transperitoneale Nierenbeckenplastik nach Anderson und Hynes durchgeführt, alle anderen Techniken wurden nicht berücksichtigt. Die Lagerung der Patienten erfolgte in Seitenlage.

**Figure 1:**
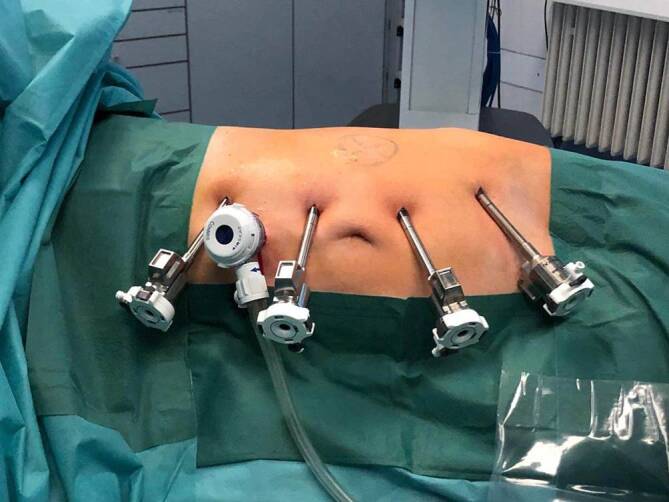

**Figure 2:**
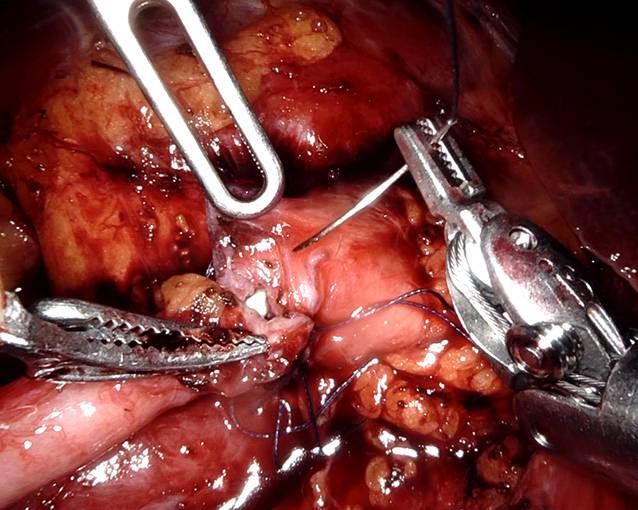

Bei der L‑NBP erfolgte eine standardmäßige Platzierung von drei Trokaren, bei der R‑NBP wurde zusätzlich ein vierter bzw. fünfter Hilfstrokar verwendet (Abb. 1). Zu Beginn wurde das Kolon nach medial mobilisiert und der Harnleiter aufgesucht. Anschließend wurde der Harnleiter mobilisiert und bis in das Nierenbecken verfolgt, dieses wurde ebenfalls freipräpariert. Im nächsten Schritt wurde die Engstelle reseziert, der Harnleiter spatuliert und das überschüssige Nierenbecken entfernt. Bei Vorliegen von Nierensteinen wurden diese nach Eröffnen des Nierenbeckens mittels flexibler Ureterorenoskopie oder bei günstiger Lage mittels Beckhoff-Klemme entfernt. Bei Vorliegen kreuzender Unterpolgefäße wurde der Harnleiter nach ventral verlagert. Die Anastomosennaht erfolgte mittels Einzelknopfnähten (Abb. 2). Vor Verschluss der Anastomose wurde ein Double‑J (DJ) eingelegt. Zuletzt wurde eine Drainage platziert, die Trokare entfernt und die Einstichstellen schichtweise verschlossen. Alle Patienten wurden mit einem Dauerkatheter versorgt.

## Statistische Methoden

Es wurden für beide Gruppen retrospektiv demographische, klinische, peri- und postoperative Informationen inklusive Komplikationen ausgewertet und verglichen. Die Komplikationen wurde entsprechend der Clavien-Dindo-Klassifikation [7] bewertet.

Kontinuierliche Daten wurden mittels Student’s t‑Test bzw. Mann-Whitney-U-Test, binäre bzw. kategorische Daten mit dem exakten Test nach Fischer bzw. χ^2^-Test verglichen. Als zweiseitiges Signifikanzniveau wurde ein Wert von 0,05 definiert. Die statistischen Berechnungen erfolgten mit SPSS Version 25.0 (SPSS, Inc., Chicago, IL).

## Ergebnisse

Insgesamt wurden im definierten Zeitraum von Mai 2015 bis September 2019 76 Nierenbeckenplastiken durchgeführt, darunter 6 offene bzw. 7 Fenger-Plastiken, welche nicht für die weitere Datenanalyse berücksichtig wurden. Es wurden somit 63 Patienten in diese Studie eingeschlossen, welche entweder eine L‑NBP oder eine R‑NBP erhielten. Die präoperativen Patientencharakteristika sind in Tab. 3 zusammengefasst.

Die durchschnittliche Laparoskopieerfahrung als Erstoperateur zum jeweiligen Zeitpunkt der L‑NBP betrug 5,22 ± 2,38 Jahre (insgesamt zehn Operateure). Zum Zeitpunkt der Einführung des Robotersystems bestand lediglich bei einem Operateur eine Vorerfahrung von ca. 50 Eingriffen mit diesem System. Die durchschnittliche Laparoskopieerfahrung als Erstoperateur zum Zeitpunkt der Einführung des Robotersystems betrug 6,09 ± 2,34 Jahre (insgesamt fünf Operateure).

Die mittlere Operationszeit war bei der R‑NBP kürzer (159 ± 54 vs. 180 ± 72 min, *p* = 0,194), der Unterschied war jedoch nicht statistisch signifikant. Eine Konversion zur offenen Technik war in keiner Gruppe notwendig. Die postoperativen Schmerzen wurden mittels der visuellen Analogskala gemessen. Es zeigte sich kein signifikanter Unterschied bzgl. der postoperativen Schmerzen (R-NPB 2,7 ± 1,9; L‑NBP 3,2 ± 2,4, *p* = 0,46). Die Länge des Krankenhausaufenthalts war in der Gruppe der R‑NBP nicht signifikant kürzer (6,33 ± 2,04 vs. 7,48 ± 2,86 Tage, *p* = 0,068).

Die postoperativen Komplikationen (während des Krankenhausaufenthalts und bis 30 Tage postoperativ) nach Clavien-Dindo wurden in Tab. 1 und 2 zusammengefasst. In Tab. 1 wurden die Komplikationen nach der Art der Komplikation aufgeteilt. Hier zeigte sich bei der L‑NBP eine signifikant höhere Rate an postoperativen Infektionen. Bei den restlichen Komplikationen, sowie nach Aufteilung in Major- und Minor-Komplikationen, ergab sich kein signifikanter Unterschied.

**Table 1:** 

Clavien-Dindo	Beschreibung	Gesamt	L‑NBP	R‑NBP	*p*
**I**	Starke Schmerzen	2 (3,2 %)	1 (3,7 %)	1 (2,8 %)	1,000
**I (ohne Revision)**	Wundinfektion, Wunddehiszenz	1 (1,6 %)	0 (0,0 %)	1 (2,8 %)	1,000
**II**	Fieberhafter Infekt, antibiotische Therapie ^a^	4 (6,3 %)	4 (14,8 %)	0 (0,0 %)	0,029
**III (Revision)**	Wundinfektion, Wunddehiszenz	1 (1,6 %)	0 (0,0 %)	1 (2,8 %)	1,000
**III**	DJ-Okklusion/Dislokation	2 (3,2 %)	0 (0,0 %)	2 (5,6 %)	0,500
**III**	Urinom (PCN-Anlage)	2 (3,2 %)	2 (7,4 %)	0 (0,0 %)	0,180
**Gesamt**	–	12 (19,0 %)	7 (25,9 %)	5 (13,9 %)	0,330

*R‑NBP* roboterassistierte Nierenbeckenplastik, *L‑NBP* laparoskopische Nierenbeckenplastik

^a^Verlängerte postoperative antibiotische Therapie abweichend vom Routineschema

**Table 2:** 

Clavien-Dindo	Gesamt	L‑NBP	R‑NPB	*p*
Minor (1 + 2)	7 (11,1 %)	5 (18,5 %)	2 (5,6 %)	0,128
Major 3	5 (7,9 %)	2 (7,4 %)	3 (8,3 %)	1,000
Major >3	0 (0,0 %)	0 (0,0 %)	0 (0,0 %)	1,000
Gesamt	12 (19,0 %)	7 (25,9 %)	5 (13,9 %)	0,332

*R‑NBP* roboterassistierte Nierenbeckenplastik, *L‑NBP* laparoskopische Nierenbeckenplastik

Zur Errechnung des Nachsorgezeitraums wurden Kontrollen im jeweiligen Krankenhaus bzw. beim betreuenden Urologen, sofern hierzu Daten vorlagen, berücksichtigt. Der mediane Nachsorgezeitraum in der Gruppe der L‑NBP war 22,5 (Range: 2,7–59,6) Monate bzw. 12,7 (Range: 0–31,7) Monate in der Gruppe der R‑NBP. Der Nachsorgezeitraum in der Gruppe der L‑NBP war signifikant länger.

Bei einem Patienten zeigte sich in der postoperativen Kontrolle (3,5 Monate nach der R‑NBP) mittels MAG3-Nierenszintigraphie eine neuerliche hochgradige Abflussbehinderung aus der betroffenen Niere. Eine neuerliche Nierenbeckenplastik wurde aufgrund der geringen verbleibenden Nierenfunktion von <20 % jedoch nicht durchgeführt (Tab. 4).

**Table 3:** 

Beschreibung	L‑NBP	R‑NBP	*p*
Patienten (*n*)	27	36	–
Alter (Jahre, MW [SD])	47,0 (20,7)	48,42 (18,4)	0,770
BMI (kg/m^2^, MW [SD])	25,6 (4,2)	24,5 (4,5)	0,376
Seite (links/rechts)	13/14	21/15	0,454
Steine (ja/nein)	6/21	6/30	0,747
Geschlecht (weiblich/männlich)	12/15	17/19	0,827

*MW* Mittelwert, *SD* Standardabweichung, *BMI* Body Mass Index, *R‑NBP* roboterassistierte Nierenbeckenplastik, *L‑NBP* laparoskopische Nierenbeckenplastik

## Diskussion

Die L‑NBP wurde für viele Jahre als neuer Goldstandard der Nierenbeckenplastik angesehen [10]. Die Langzeitergebnisse sind mit denen der offenen Nierenbeckenplastik vergleichbar und weisen eine geringere Morbidität auf [5, 11, 15].

Mittlerweile liegt auch eine Vielzahl an Daten vor, dass die R‑NBP sehr gute Ergebnisse liefert [2, 15]. In einem systematischen Review von 2009 konnte kein Unterschied zwischen L‑NBP bzw. R‑NBP in Bezug auf postoperative Urinome, Erfolgsraten und Operationszeiten gezeigt werden. Es zeigte sich lediglich eine signifikant kürzere Krankenhausaufenthaltsdauer in der Gruppe der R‑NBP [4]. In diesem systematischen Review wurden allerdings Studien mit sehr kleinen Fallzahlen und geringer Qualität eingeschlossen. Darauf folgende Studien zeigten ähnliche Ergebnisse ([3, 9, 12]; Tab. 4).

**Table 4:** 

Beschreibung	L‑NBP	R‑NBP	*p*
Patienten (*n*)	27	36	–
Operationszeit (min MW [SD])	180 (72)	159 (54)	0,194
Konversion zur offenen Operation	0/32 (0,0 %)	0/32 (0,0 %)	1,000
Postoperative VAS (Median Range)	2,9 (0–8)	2,7 (0–7)	0,717
Krankenhausaufenthalt (Tage MW [SD])	7,48 (2,86)	6,33 (2,04)	0,068
Relevante Restenose	0 (0,0 %)	1 (2,8 %)	1,000
Nachsorgezeitraum (Monate Median [Range])	22,5 (2,7–59,6)	12,7 (0–31,7)	0,003

*MW* Mittelwert, *SD* Standardabweichung, *R‑NBP* roboterassistierte Nierenbeckenplastik, *L‑NBP* laparoskopische Nierenbeckenplastik, *VAS* Visuelle Analogskala

Bei den meisten Studien wurden zwei etablierte Systeme verglichen bzw. isoliert die Ergebnisse der R‑NBP betrachtet. In einer prospektiven Studie aus 2006 ergaben sich signifikant längere Operationszeiten für die Gruppe der R‑NBP [13]. Das Andocken des Robotersystems bzw. das Setzen der Trokare zählt bereits zur Operationszeit. In einigen rezenten Studien zeigte sich bereits der Trend von kürzeren Operationszeiten in dieser Gruppe. Ein signifikanter Unterschied ergab sich jedoch in kaum einer Studie [2]. In unserer retrospektiven Studie war die Operationszeit in der Gruppe der R‑NBP kürzer, jedoch nicht signifikant (159 ± 54 vs. 180 ± 72 min, *p* = 0,194). Bei den eingeschlossenen Studien einer Metaanalyse von Autorino et al. 2014 wurden mediane Operationszeiten von 98–312 min in der R‑NBP bzw. 81–324 min in der L‑NBP-Gruppe angegeben [2]. Vor allem bei den ersten Operationen ist das Andocken des Roboters relativ zeitaufwändig. Dies kann jedoch durch das schnellere Nähen der Anastomose bei der R‑NPB wieder ausgeglichen werden. Durch ein gut eingespieltes Team kann das Andocken im weiteren Verlauf wesentlich schneller durchgeführt werden, wodurch sich möglichweise kürzere Operationszeiten ergeben könnten.

Bei der Datenanalyse zeigte sich in der L‑NBP-Gruppe eine signifikant höhere Rate an postoperativen Infektionen (0 vs. 14,8 %, *p* = 0,029), welche antibiotisch behandelt wurden. Dieser signifikante Unterschied ist unserer Meinung nach nicht durch die Methodik bedingt, sondern könnte u. a. durch die verbesserte präoperative Antibiotikastrategie zu erklären sein. In einem Artikel von Rai 2016 konnte bereits gezeigt werden, dass die Einhaltung der EAU-Richtlinien bzgl. präoperativer Antibiotikaprophylaxe zu einem reduzierten Einsatz von Antibiotika führt, ohne dabei die Anzahl an postoperativen Infektionen zu erhöhen [6].

In einer Metaanalyse 2018 zeigten sich signifikant weniger Krankenhausaufenthaltstage in der R‑NBP-Gruppe [11]. Auch bei unseren Daten zeigten sich kürzere Aufenthaltsdauern der Patienten (7,48 vs. 6,33, *p* = 0,068). In unserem Fall scheint die unterschiedliche Verweildauer sicherlich auch durch ökonomische bzw. strategische Überlegungen beeinflusst zu sein.

Es ergab sich kein signifikanter Unterschied bei der Rate an Minor- bzw. Major-Komplikationen. In der Gruppe der R‑NBP wurde bei einem Patienten eine relevante Restenose diagnostiziert. Insgesamt ergibt sich eine sehr hohe Erfolgsrate für beide Gruppen. Bei diesen Daten müssen natürlich die unterschiedlich langen Nachsorgezeiträume aufgrund des Designs der Studie berücksichtig werden. Da die R‑NBP zeitlich nach der L‑NBP durchgeführt wurde, ergeben sich auch kurze Nachsorgezeiträume für die robotische Gruppe. Aufgrund der geringen Rezidivrate von 0–8 % [2] wäre eine wesentlich größere Fallzahl notwendig, um verlässlich signifikante Unterschiede zu detektieren.

Bei dieser Studie wurde eine retrospektive Analyse, basierend auf medizinischen Unterlagen bzw. Berichten, durchgeführt, wodurch einige Limitationen entstehen. Für die Indikationsstellung und für die Nachsorge gab es kein standardisiertes Vorgehen. Standardisierte und validierte Fragebögen in der Nachsorge würden die Beurteilung einer erfolgreichen Operation wesentlich verbessern.

Der Trend zur Roboterchirurgie scheint sich weiter fortzusetzen und viele Krankenhäuser implementieren roboterassistierte Systeme. Die Einführung dieser Systeme führt in den meisten Fällen auch zu einer positiven Werbewirkung für die entsprechende Abteilung. Wie sich das peri- bzw. postoperative Outcome unmittelbar nach Einführung dieser Systeme verändert, wurde bisher kaum untersucht. Unsere retrospektive Studie konnte zeigen, dass unmittelbar nach Einführung der roboterassistierten Chirurgie bereits vergleichbar gute Ergebnisse erzielt werden können und kein Nachteil für den Patienten entsteht.

Mittlerweile stellt die R‑NBP die Standardoperation in beiden Zentren dar. Der große Vorteil der Robotik ist nach dem ablativen Teil, der rekonstruktive Part mit dem schnellen und präzisen Nähen der zusätzlich fast wasserdichten Anastomose. Die Frage, ob bei der Präzision durch die Robotik noch eine DJ-Schienung notwendig ist, scheint gerechtfertigt und kann abschließend nur im Rahmen einer eigenen Studie beantwortet werden. So kommen wir wiederum auf die Anfänge zurück, da auch im Rahmen der Erstbeschreibung durch Anderson und Hynes auf eine DJ-Anlage, wann immer möglich, verzichtet werden sollte, da diese zu weiteren Irritationen und aufsteigenden Infektionen führen kann.

## Schlussfolgerung

Unsere Daten zeigen keinen signifikanten Unterschied der beiden Gruppen bezogen auf die peri- und postoperativen Ergebnisse. Es konnte gezeigt werden, dass für den Patienten auch unmittelbar nach Implementierung eines robotischen Systems kein Nachteil entsteht.

Zur besseren Beurteilung von Langzeitergebnissen sind weitere Studien mit definierten Nachsorgeuntersuchungen bzw. Intervallen notwendig. Außerdem würden standardisierte und validierte Fragebögen, sowie standardisierte Kontrolluntersuchungen in der Nachsorge die Beurteilung einer erfolgreichen Operation wesentlich verbessern.

## References

[1] Anderson JC, Hynes W (1949). RETROCAVAL URETER:A Case diagnosed pre-operatively andtreated successfully by a Plastic Operation. Br J Urol.

[2] Autorino R, Eden C, El-Ghoneimi Alaaand Guazzoni G (2014). Robot-assisted and laparoscopic repair of ureteropelvic junctionobstruction: a systematic review and meta-analysis. Eur Urol.

[3] Bird VG, Leveillee RJ, Eldefrawy A (2011). Comparison of robot-assisted versus conventional laparoscopictransperitoneal pyeloplasty for patients with ureteropelvicjunction obstruction: a single-center study. Urology.

[4] Braga LHP, Pace K, DeMaria J, Lorenzo AJ (2009). Systematic review and meta-analysis of robotic-assisted versusconventional laparoscopic pyeloplasty for patients withureteropelvic junction obstruction: effect on operative time,length of hospital stay, postoperative complications, and successrate. Eur Urol.

[5] Brooks JD, Kavoussi LR, Preminger GM (1995). Comparison of open and endourologic approaches to the obstructedureteropelvic junction. Urology.

[6] Cai T, Verze P, Brugnolli A (2016). Adherence to European Association of Urology Guidelines on prophylactic antibiotics: an important step in antimicrobial stewardship. Eur Urol.

[7] Dindo D, Demartines N, Clavien P-A (2004). Classification of surgical complications: a new proposal withevaluation in a cohort of 6336 patients and results of a survey. Ann Surg.

[8] Gettman MT, Neururer R, Bartsch G, Peschel R (2002). Anderson-Hynes dismembered pyeloplasty performed using the da Vinci robotic system. Urology.

[9] Hong P, Ding G, Zhu D (2018). Head-to-head comparison of modified laparoscopic pyeloplasty and robot-assisted pyeloplasty for ureteropelvic junction obstruction in China. Urol Int.

[10] Janetschek G, Peschel R, Frauscher F (2000). Laparoscopic pyeloplasty. Urol Clin North Am.

[11] Kolontarev K, Kasyan G, Pushkar D (2018). Robot-assisted laparoscopic ureteral reconstruction: а systematicreview of literature. Cent European J Urol.

[12] Kumar R, Nayak B (2013). Robotic versus conventional laparoscopic pyeloplasty: a singlesurgeon concurrent cohort review. Indian J Urol.

[13] Link RE, Bhayani SB, Kavoussi LR (2006). A prospective comparison of robotic and laparoscopic pyeloplasty. Ann Surg.

[14] Rashid TG, Kini M, Ind TEJ (2010). Comparing the learning curve for robotically assisted and straight stick laparoscopic procedures in surgical novices. Int J Med Robot Comput Assist Surg.

[15] Traumann M, Kluth LA, Schmid M (2015). Roboterassistierte laparoskopische Pyeloplastik bei Erwachsenen. Urologe.

[16] Williams B, Tareen B, Resnick MI (2007). Pathophysiology and treatment of ureteropelvic junction obstruction. Curr Urol Rep.

[17] Yohannes P, Rotariu P, Pinto P (2002). Comparison of robotic versus laparoscopic skills: is there adifference in the learning curve?. Urology.

